# Mycoplasma non-coding RNA: identification of small RNAs and targets

**DOI:** 10.1186/s12864-016-3061-z

**Published:** 2016-10-25

**Authors:** Franciele Maboni Siqueira, Guilherme Loss de Morais, Susan Higashi, Laura Scherer Beier, Gabriela Merker Breyer, Caio Padoan de Sá Godinho, Marie-France Sagot, Irene Silveira Schrank, Arnaldo Zaha, Ana Tereza Ribeiro de Vasconcelos

**Affiliations:** 1 Centro de Biotecnologia (CBiot), Universidade Federal do Rio Grande do Sul (UFRGS), Porto Alegre, Rio Grande do Sul Brazil; 2 Laboratório Nacional de Computação Científica (LNCC), Laboratório de Bioinformática (LABINFO), Petrópolis, Rio de Janeiro Brazil; 3 Inria Grenoble Rhône-Alpes, 38330 Montbonnot Saint-Martin, France; 4 Université Lyon 1, Villeurbanne, France; 5 CNRS, UMR5558, Laboratoire de Biométrie et Biologie Évolutive, F-69622 Villeurbanne, France

**Keywords:** *Mycoplasma hyopneumoniae*, *Mycoplasma flocculare*, *Mycoplasma hyorhinis*, Small RNAs, Porcine respiratory system

## Abstract

**Background:**

Bacterial non-coding RNAs act by base-pairing as regulatory elements in crucial biological processes. We performed the identification of trans-encoded small RNAs (sRNA) from the genomes of *Mycoplama hyopneumoniae*, *Mycoplasma flocculare* and *Mycoplasma hyorhinis*, which are *Mycoplasma* species that have been identified in the porcine respiratory system.

**Results:**

A total of 47, 15 and 11 putative sRNAs were predicted in *M. hyopneumoniae*, *M. flocculare* and *M. hyorhinis*, respectively. A comparative genomic analysis revealed the presence of species or lineage specific sRNA candidates. Furthermore, the expression profile of some *M. hyopneumoniae* sRNAs was determined by a reverse transcription amplification approach, in three different culture conditions. All tested sRNAs were transcribed in at least one condition. A detailed investigation revealed a differential expression profile for two *M. hyopneumoniae* sRNAs in response to oxidative and heat shock stress conditions, suggesting that their expression is influenced by environmental signals. Moreover, we analyzed sRNA-mRNA hybrids and accessed putative target genes for the novel sRNA candidates. The majority of the sRNAs showed interaction with multiple target genes, some of which could be linked to pathogenesis and cell homeostasis activity.

**Conclusion:**

This study contributes to our knowledge of *Mycoplasma* sRNAs and their response to environmental changes. Furthermore, the mRNA target prediction provides a perspective for the characterization and comprehension of the function of the sRNA regulatory mechanisms.

**Electronic supplementary material:**

The online version of this article (doi:10.1186/s12864-016-3061-z) contains supplementary material, which is available to authorized users.

## Background

In bacterial genomes the non-coding RNAs (ncRNA) identified were the ribosomal RNAs (rRNA) and transfer RNAs (tRNA), which are important components of the protein synthesis machinery [1–3]. In addition, cis-encoded antisense RNAs (asRNA) and trans-encoded small RNAs (sRNA) were also identified. Analyses of asRNAs and sRNAs targets have shown that these ncRNAs could alter the translation process or mRNA stability by target base pairing [3–6]. Moreover, sRNAs may have multiple targets [4, 5].

Non-coding RNA elements present in many bacterial genomes add a further complexity to the comprehension of bacterial gene regulation [7]. Recently, several ncRNAs with different genomic origins, lengths, functions, and gene regulation mechanisms have been identified [6, 8–10]. There are evidences that ncRNAs may regulate important processes, such as pathogenesis, iron metabolism, and quorum sensing [4, 11, 12].

Novel ncRNAs are difficult to detect by conventional biochemical screenings [13]. As an alternative, *in silico* approaches associated to a functional analysis validation have proved to be effective in the identification of ncRNAs [14–17]. In AT-rich genomes, the ncRNA genes show a relatively higher GC-content [18] and therefore, compositional-based analyses that scan for local GC-content have had success in detecting ncRNAs. The algorithm Single Genome ncRNA Search (SIGRS) [19] is a whole-genome eukaryote predictor that uses these features. Given that most functional RNAs rely on a stable secondary structure, prediction of the minimum free energy of a transcript is also used as a means of detecting ncRNA genes [20].

Target prediction is the main step in understanding bacterial sRNA function. Recently, computational target prediction methods had their performance improved by inclusion of RNA accessibility and conservation information [21–24]. Interacting RNA (IntaRNA) and RNAplex are reliable sRNA target prediction software [25]. IntaRNA uses the energy score of the interaction, which is calculated as the sum of the free energy of hybridization and the free energy required for making the interaction sites accessible [22]. RNAplex [21] is a refinement of the RNAhybrid software and uses a simplified algorithm to reduce the time needed to localize putative hybridization sites, mainly by neglecting intramolecular interactions and by using a slightly simplified energy model. The RNAplex tool is combined with the RNAup tool and can find high-confidence targets, with only a slight loss of sensitivity. Moreover, RNAplex also uses an energy score of the interaction of sRNA and putative targets to predict molecule interactions.

Mycoplasmas are bacteria of the class Mollicutes characterized by small genomes and low GC content. *Mycoplasma hyopneumoniae*, *Mycoplasma flocculare* and *Mycoplasma hyorhinis* are important species that have been identified in the porcine respiratory system [26–28]. *M. hyopneumoniae* is the etiological agent of porcine enzootic pneumonia [29], while *M. hyorhinis* can also cause swine polyserositis and arthritis [30]. Although *M. flocculare* is widespread in swine herds, it has so far been recognized as a commensal species [31]. Currently, the genome sequences of several *Mycoplasma* species are available, allowing a comparative analysis of the gene content among different species. However, information related to regulatory elements is very limited in these organisms. Furthermore, the *Mycoplasma* species present a low number of proteins involved in transcriptional regulation [32, 33]. These evidences suggest the presence of alternative transcriptional regulatory mechanisms in mycoplasmas.

It was previously shown that most genes from the genomes of *M. hyopneumoniae*, *M. flocculare* and *M. hyorhinis* are expressed at some basal level and that the majority of the genes are co-transcribed [34, 35]. Therefore, a global determination of the genomic functional elements is a prerequisite to expand our knowledge regarding transcriptional small RNA regulation in swine respiratory mycoplasmas.In the current study, we have analyzed and predicted trans-encoded small RNAs from *M. hyopneumoniae*, *M. flocculare* and *M. hyorhinis* genomes. Moreover, we have analyzed RNA-RNA interaction and accessed target genes for the sRNA candidates. Some predicted *M. hyopneumoniae* sRNAs were also experimentally investigated by a reverse transcription amplification approach in three different culture conditions.

## Results

### Global screening for small RNAs

Knowledge related to the presence and role of small RNAs in mycoplasma remains limited, therefore a genome-wide screen for sRNAs was conducted using *in silico* prediction approaches. Only sRNAs present in the intergenic regions (IGRs) were searched as the input file, since all regions marked as coding sequence (CDS) and known ncRNAs, such as rRNA, tRNA, RNase P and others, were masked. These IGRs have a GC content of 21 % for *M. hyopneumoniae*, 23 % for *M. flocculare* and 21 % for *M. hyorhinis* representing, respectively, 13, 12 and 14 % of the total genome. The segments with a high GC cumulative, comparable to the known ncRNAs, were considered sRNA candidates.

The analysis of the *M. hyopneumoniae* genome using the SIGRS software allowed the identification of 17 previously known ncRNAs and 26 predicted putative novel sRNAs. However, the SIGRS outputs associate with distinct regions, defined some of them as unique ncRNA candidates. Therefore, a fragmentation algorithm (FraPS) was applied, resulting in 25 previously known ncRNAs (representing 71 % of the recovered input) and 47 putative novel sRNAs, named sRNA_1 to sRNA_47 (Additional file 1: Table S1). The sequences of these predicted sRNAs including their information related to genome location, length, GC content, and free energy of the secondary structure of the sRNA are supplied in Additional file 2: Table S2. The RNAspace software recovered 87 % of the SIGRS/FraPS predictions supporting the previous SIGRS/FraPS results for the sRNA genes.

*M. hyopneumoniae* sRNAs have an average length of 128 bp, ranging from 61 to 424 bp and 40.8 % of GC content. Furthermore, the minimum fold energy (∆G) among the putative sRNAs varies from −0.057 kcal/mol to −0.296 kcal/mol (Additional file 2: Table S2). The sRNA sequences were analyzed by Blast search to locate homologous sequences in the genomes of the other *M. hyopneumoniae* strains available. Remarkably, 35 predicted novel sRNAs (74 %) were present in all *M. hyopneumoniae* genome strains (J, 7422, 232, 168 and 168-L), while only the sRNA_42 was exclusive to the *M. hyopneumoniae* 7448 genome (see Additional file 1: Table S1). Interestingly, only the sRNA_31 was identified in the genomes of all pathogenic strains and was absent in the genome of the non-pathogenic *M. hyopneumoniae* J strain. Moreover, sRNA_07 was found only in the genome of the two pathogenic strains 7448 and 7422 isolated from Brazilian swine herds.

**Fig. 1:**
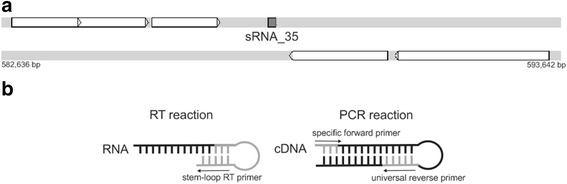
Schematic representation of sRNA position at *M. hyopneumoniae* 7448 genome and primers design. **a** Overview of the location of sRNA_35 in the *M. hyopneumoniae* 7448 genome region from 582,636 to 593,642 base pair. *White arrows* represent the genes position and the sRNA_35 is represented by a *gray box*. Representation from Artemis. **b** Primers location for the RT-PCR stem-loop reaction. *Slim arrows* indicate the amplicon orientation. *Black* portions represent amplification and *gray* portions represent the primers location. The amplification from specific forward primer to universal reverse primer is the entire sRNA length

In order to evaluate if some of the predicted sRNA genes were transcribed, a stem-loop-RT-PCR analysis was used to investigate the presence of sRNA transcripts in three different culture conditions. Figure 1b illustrates the primers design for the stem-loop-RT-PCR approach. Primer pair positions were based on a full-length sRNA prediction, for both strands using specific primers for each sRNA tested. The low GC content of the *M. hyopneumoniae* genome and the mandatory full-length primers position with high specificity resulted in 19 sRNAs subject to experimental analysis. It was possible to show that all the 19 predicted and experimentally analyzed sRNAs were detected as transcribed in the three different growth conditions, except for sRNA_05 and sRNA_09, which were not transcribed in the standard condition or heat shock condition, respectively (Additional file 3: Table S3).

The possible presence of sequences homologous to the novel sRNAs predicted for *M. hyopneumoniae* in other *Mycoplasma* species, also found in the swine respiratory tract, was analyzed by Blast search. Sequences homologous to the novel sRNAs of *M. hyopneumoniae* were not found in the genomes of *M. flocculare* and *M. hyorhinis*, supporting the notion that these novel sRNAs are species-specific. Therefore, the genomes of *M. flocculare* and *M. hyorhinis* were screened for the presence of sRNAs applying the same methodology used for *M. hyopneumoniae*.

Using SIGRS plus FraPS, it was possible to predict 15 novel sRNAs for *M. flocculare*, which were named sRNA_F1 to sRNA_F15 (Additional file 4: Table S4) and 11 novel sRNAs for *M. hyorhinis*, which were named sRNA_R1 to sRNA_R11 (Additional file 5: Table S5). RNAspace predictor was able to identify approximately 50 % of the SIGRS/FraPS predictions, for both genomes. Furthermore, the main profile of the new predicted sRNAs was similar to that found for the sRNAs predicted in *M. hyopneumoniae*. The sRNAs of *M. flocculare* have an average length of 167 bp, ranging from 76 to 402 bp and 36.7 % of GC content (Additional file 6: Table S6), while the minimum fold energy (∆G) among the putative sRNAs varies from −0.063 kcal/mol to −0.493 kcal/mol (Additional file 6: Table S6). The sRNAs of *M. hyorhinis* have an average length of 85 bp, ranging from 35 to 209 bp with 44.4 % of GC content and the minimum fold energy (∆G) varied from −0.123 kcal/mol to −0.392 kcal/mol (Additional file 7: Table S7).

A homologous sequence search was carried out for each novel putative *M. flocculare* and *M. hyorhinis* sRNA in the available genomes of other *M. flocculare*, *M. hyorhinis* and *M. hyopneumoniae* strains. All predicted sRNAs were present only in the corresponding genomes of *M. flocculare* (strain 27399) or *M. hyorhinis* (strains DBS1050, MCLD and SK76), as shown in Additional file 4: Table S4 and Additional file 5: Table S5.

### Small RNA targets prediction

The importance of sRNAs as a distinct class of gene regulators in bacteria is well established, as many diverse processes have been shown to be controlled by sRNAs in different species [4, 11, 12]. The interaction of sRNAs with different targets is an important mechanism to control the complex regulatory networks in bacterial cells. Therefore, to analyze the interaction of sRNAs to a multitude of different target mRNAs and their role in gene regulation, the binding region of the sRNA:target duplex needs to be investigated.

A computational analysis of the potential targets for the sRNAs identified using RNAplex and IntaRNA predicted 204 targets for the sRNAs of *M. hyopneumoniae* (see Additional file 1: Table S1 and Additional file 8: Table S8), 35 targets for the sRNAs of *M. flocculare* (see Additional file 4: Table S4 and Additional file 9: Table S9) and 42 targets for the sRNAs of *M. hyorhinis* (see Additional file 5: Table S5 and Additional file 10: Table S10). An interaction was deemed functional only if respecting both criteria free energy smaller or equal to a threshold of −13 kcal/mol and equally predicted by the two software. Detailed information (i.e., gene ID, gene name, gene product and interaction energy - kcal/mol) related to the sRNAs/targets interactions is available in each Additional file.

The *in silico* predictions identified interaction with target genes for 44 novel *M. hyopneumoniae* sRNAs. The majority of the sRNAs showed multiple target genes varying from 1 to 26 (Additional file 1: Table S1), however most of them (90 %) interacted with up to seven target genes (Additional file 1: Table S1). Considering the whole genome of *M. hyopneumoniae*, 145 genes out of 678 showed a predicted interaction with at least one novel sRNA, representing 21 % of the *M. hyopneumoniae* genes used as input (Additional file 8: Table S8). In general, the genes that showed such predicted interaction were also found to pair with multiple sRNAs, although some genes interact with only a single sRNA.

From the 145 genes, 108 (74 %) were found as single target, meaning that the mRNAs encoded by these genes could potentially interact with only one of the predicted novel sRNAs (Additional file 8: Table S8). The products of these genes are mainly related to basal cell metabolism, transport system and lipoproteins; many products are related to protein synthesis while most are related with unknown products (hypothetical proteins). A detailed analysis of the data (Additional file 8: Table S8) revealed that approximately 25 % of the target genes were predicted as capable to interact with up to three sRNAs. Nevertheless, some targets are ubiquitous: for example the gene AAZ53944.1, which encodes the exonuclease protein, showed interactions with six predicted sRNAs. Another gene with multiple sRNA interactions is AAZ54018.1 gene, which codes for the prolipoprotein p65, which may interact with three different sRNAs.

The three novel sRNAs without target prediction were sRNA_27, sRNA_28 and sRNA_37. Detailed analysis of the interaction predictions revealed that these sRNAs have putative target genes found by the two algorithms, however the energy interaction in the IntaRNA software was higher than −13 kcal/mol. The sRNA_37, experimentally analyzed, has two putative target genes: MHP7448_0312, encoding glycine cleavage system H protein, and the MHP7448_0704, encoding a hypothetical protein, with energy interactions around −12 kcal/mol for both of them. In turn, the sRNA_27 and sRNA_28 interact with targets with energy around −10 kcal/mol as: the MHP7448_0293 (ychF GTP binding protein) and MHP7448_0166 (ribosomal large subunit pseudouridine synthase B) as targets for sRNA_27; and MHP7448_0362 (ABC transport system permease protein p69-like), MHP7448_0022 (hypothetical protein) and MHP7448_0601 (hypothetical protein) as targets for sRNA_28 (Additional file 8: Table S8).

Similarly to the *M. hyopneumoniae* results, RNAplex and IntaRNA predicted target gene interactions for 13 novel *M. flocculare* sRNAs and for all novel *M. hyorhinis* sRNAs. Most of the sRNAs were predicted to bind to multiple targets and the number of targets for each sRNA ranged from one to nine for *M. flocculare* and from one to 13 for *M. hyorhinis* (Additional file 9: Table S9 and Additional file 10: Table S10). As shown in Additional file 4: Table S4 and Additional file 5: Table S5, the majority of the predicted novel sRNAs from both genomes were able to interact with a restricted number of target genes. The sRNAs with the higher interaction number were the sRNA_F7 and the sRNA_R6, with 13 and 9 target interactions, respectively.

Data analyses, as presented in Additional file 9: Table S9 and Additional file 10: Table S10, showed that 33 of the encoded mRNAs of *M. flocculare* and 41 of the encoded mRNAs of *M. hyorhinis* interact with at least one novel sRNA, representing, respectively, 5 and 6 % of all the genes used as input. As expected, the different products of the target genes have different metabolic functions as observed in the *M. hyopneumoniae* analysis. Some of the genes encode for enzymes involved in cell metabolism, cell division proteins, transcription regulation, adhesins, while a high number of the gene products are classified as unknown or hypothetical proteins.

The *M. flocculare* sRNAs without target prediction were sRNA_F5 and sRNA_F15. Detailed analysis of the interaction predictions shows these sRNAs with putative target genes found by the two algorithms, however the energy interaction in the IntaRNA software was higher than −13 kcal/mol. Focusing in these *in silico* interactions, the sRNA_F5 was able to bind to genes *rpsk* (30S ribosomal protein S11), *trxA* (thioredoxin), and MF01218 (P37-like ABC transporter substrate binding lipoprotein). Furthermore, sRNA_F15 could interact with MF01377 and MF01379 (both hypothetical proteins) and also with MF00736 (ABC transport permease protein). The interaction energies for both sRNAs were around −10 kcal/mol.

## Discussions

Small bacterial RNAs generally act by base pairing with mRNAs, regulating many aspects of bacterial physiology leading to positive or negative regulation of target protein synthesis. To search for sRNAs in the genomes of *M. hyopneumoniae*, *M. flocculare* and *M. hyorhinis*, we combined *in silico* prediction approaches with transcription analysis (RT-PCR).

The *in silico* prediction of novel trans-acting sRNAs in the three mycoplasma genomes was performed by applying two different methods, ncRNA Search (SIGRS) and RNAspace, allowing the *in silico* identification of sRNAs following the combination of the results generated by both algorithms. Using this approach, 47, 15 and 11 putative novel sRNAs were predicted in the genomes of *M. hyopneumoniae*, *M. flocculare* and *M. hyorhinis*, respectively. The average length of these putative sRNAs in the genomes of the three mycoplasma species ranged from 85 to 167 bp. Previously, the availability of transcriptome maps of *M. hyopneumoniae*, *M. flocculare* and *M. hyorhinis* allowed the *in silico* identification of 78, 130 and 72 putative novel ncRNAs, respectively, with lengths ranging from 30 to 600 nucleotides [35]. However, this approach was unable to identify the sRNAs among the ncRNAs [35]. Although in *Escherichia coli* the majority of the new sRNAs, varying in length from 50 to 400 nucleotides, have been identified through an *in silico* prediction [36, 37], the application of this proposed methodology to other bacterial species has had only limited success as it requires reliable species-specific consensus promoter and terminator sequences. In *Vibrio cholerae*, 32 candidates for novel sRNAs were predicted by relying only on putative terminators and regions of sequence conservation in intergenic regions [38]. Previous studies on the *M. pneumoniae* genome demonstrated the presence of 311 ncRNAs, the majority of which were classified as asRNAs, and probably only 19 are sRNAs [10, 39]. Among other bacteria, the number of sRNAs is variable as 83 trans-encoded sRNAs found in the *Listeria monocytogenes* genome [12, 40–44]. Taken together, it is possible to suggest that the number and size of the sRNAs predicted in the genomes of *M. hyopneumoniae*, *M. flocculare* and *M. hyorhinis* are variable as those found in other bacterial species.

Aiming to validate the *in silico* sRNA prediction, some *M. hyopneumoniae* sRNAs were amplified. Nineteen out of 47 predicted sRNAs were tested, corresponding to all candidates that could be full-length amplified by stem-loop RT specific primers. The experimental data were able to validate the *in silico* approach as transcripts were detected from all tested sRNAs. Interestingly, two of the sRNAs (sRNA_05 and sRNA_09) showed differential expression dependent on the growth conditions tested. The sRNA_05 was transcribed in both stress conditions tested and the presence of the sRNA_09 transcript was detected only in normal culture and oxidative stress conditions. These results support the notion that the expression of regulatory RNAs (such as sRNAs) changes in response to external stimuli and therefore contributes to an adaptive expression program. Moreover, different studies have also established the indispensable nature of bacterial sRNAs in cell adaptation, immediate responses to changing environments, survival, and pathogenesis [45, 46].

No homologs of the 47 *M. hyopneumoniae* sRNAs were found in the genomes of the other swine respiratory tract *Mycoplasma* species. The same result was observed when sRNAs from *M. flocculare* and *M. hyorhinis* were analyzed. These results demonstrate that the sRNAs of these three swine respiratory tract Mycoplasmas are species-specific. We have looked for any difference that could be associated to the non-conservation among species. However, the genome composition, including GC content within the coding and non-coding regions is very similar in *mycoplasmas* species. Moreover, the species-specific sRNAs were identified in the genomes of the different strains of each species analyzed. Previous studies have demonstrated that, although some sRNAs are conserved in closely related species, a similar species-specific sRNA pattern appears to be found in other bacterial species [14, 36, 37, 47, 48].

The number of regulatory RNAs predicted in *M. hyopneumoniae*, *M. flocculare* and *M. hyorhinis* indicates that many diverse processes could be controlled by bacterial sRNAs. In order to predict targets for the novel sRNAs, two algorithms were used. A genome-wide COG analysis of sRNA–mRNA target interactions demonstrated the absence of correlation to specific classes of genes, suggesting that sRNAs might be used to control general processes within the mycoplasma cell as found in other bacteria [44]. Moreover, we observed that the ortholog target genes predicted for a *Mycoplasma* species could interact with another species; so, target genes could be considered as not species-specific.

Similarly to data previously described for other bacteria, some *M. hyopneumoniae*, *M. flocculare* and *M. hyorhinis* sRNAs have a predicted interaction with single targets (e.g., sRNA_02 from *M. hyopneumoniae*, sRNA_F2 from *M. flocculare* and sRNA_R1 from *M. hyorhinis*) while others show a multitude of targets (e.g., sRNA_06 from *M. hyopneumoniae*, sRNA_F7 from *M. flocculare* and sRNA_R6 from *M. hyorhinis*), possibly acting as global regulators. Taken together, our results indicate that the majority of the predicted mRNA targets are encoded hypothetical proteins or genes involved in the cellular general metabolism.

Previous analyses described the presence of differential transcription in the *M. hyopneumoniae* transcriptome [35, 49–52]. Therefore, the genes up or down-regulated in the above studies were correlated with the genes predicted as interacting with sRNAs. Among all differentially expressed genes [49–52], 24 (20 %) were predicted as target genes for at least one novel sRNA. The gene MHP7448_0656 that encode the p65 prolipoprotein, which can interact with three different sRNAs (sRNA_10, sRNA_20 and sRNA_26) have been shown to be differentially expressed in heat chock condition [50]. p65 is an immunodominant surface lipoprotein of *M. hyopneumoniae* used in the serological diagnosis of infections [35]. Moreover, the gene MHP7448_0487 that encodes a putative MgtE transporter and is also responsive to heat chock stress [50] showed putative interaction with three different sRNAs (sRNA_7, sRNA_20 and sRNA_29). MgtE is a magnesium transporter protein expressed in a number of Gram-negative and Gram-positive bacteria that can modulate bacteria virulence and cytotoxicity [53].

Homologous sequences for the small RNAs were searched in the genomes of other *M. hyopneumoniae* strains available. Interestingly, only one sRNA (sRNA_31) was exclusive of the genomes of pathogenic strains of *M. hyopneumoniae* (Additional file 1: Table S1). The sRNA_31 interacts *in silico* with two mRNAs encoding hypothetical products and one mRNA, which encodes a protoporphirogen oxidase that has methylase activity. So, further analysis will be required to characterize the hypothetical proteins and identify a possible function for this exclusive sRNA of *M. hyopneumoniae*.

## Conclusions

In the current study, we have analyzed and predicted trans-encoded small RNAs from the genomes of *M. hyopneumoniae*, *M. flocculare* and *M. hyorhinis*. Moreover, we have analyzed RNA-RNA interaction and accessed putative target genes for the sRNA candidates. Some predicted *M. hyopneumoniae* sRNAs were also experimentally investigated by a reverse transcription amplification approach, in three different culture conditions. In conclusion, we were able to identify 47, 15 and 11 novel sRNAs in *M. hyopneumoniae*, *M. flocculare* and *M. hyorhinis*, respectively. The number of sRNAs is similar to the one predicted in other bacterial species. All *M. hyopneumoniae* sRNAs tested were transcribed in at least one condition; however, the differential expression profile of two sRNAs in response to oxidative stress and heat shock stress suggests that its expression is influenced by environmental signals. Target genes for the novel sRNA candidates were accessed showing that many sRNAs can interact with different targets, and that different sRNAs could regulate the same mRNAs. In this context, complex global regulatory networks might be implicated in *Mycoplasma*.

## Methods

### In silico analysis of small RNAs

The prediction of sRNAs was performed in *M. hyopneumoniae* 7448 (NSDC AE017244.1), *M. hyorhinis* HUB-1 (INSDC CP002170.1) and *M. flocculare* ATCC 27716 (INSDC AFCG00000000.1) using two software: Single Genome ncRNA Search (SIGRS) [19] and RNAspace [54]. SIGRS is a nucleotide contrast-based tool, which screens an input genome and indicates regions with similar nucleotide composition to known ncRNA sequences. SIGRS computes a scoring scheme that allows the transformation of the nucleotide genome sequence into a numeric one. Subsequences that aggregate a partial sum above a significant threshold are considered ncRNA gene candidates. RNAspace uses a similar strategy, screening for rich atypical GC regions, and only the regions with a GC value above the mean plus twice the standard deviation for the whole genome are considered as atypical. This method detects signals intrinsic to ncRNAs, differentiating them from other elements in the genome by exploiting the compositional bias between ncRNAs and other regions of the genome.

Initially, the SIGRS method was used as the *Mycoplasmas* sRNA predictor with a set of known ncRNAs to guide the search for new ncRNAs with a similar nucleotide composition profile and structural-based features. Known ncRNA sequences of *Mollicutes* (rRNA, tRNA, RNase P and other functional ncRNAs) were obtained from the Bacterial sRNA Database (BSRD) [55] and from the bacterial genome annotation. The genomes used for the new sRNAs search were masked in all regions marked as coding sequence (CDS) in the annotation files of *M. hyopneumoniae*, *M. flocculare* and *M. hyorhinis*. Therefore, the search was performed within the intergenic regions, which potentially harbor the sRNAs. The set of known ncRNAs and the masked genomes were provided as input to SIGRS, which creates a scoring system based on the nucleotide composition of the known ncRNAs to transform the genome in a numerical sequence. The segments with a high cumulative sum are thus considered as sRNA candidates. However, SIGRS was unable to distinguish different ncRNA candidates when the distance between them was too small. To solve this problem, a segmentation of the numerical sequence that represents the ncRNA candidates was required in order to identify the largest local slopes in a given sequence. Therefore, the algorithm by Kadane [56] to find these slopes was adapted, allowing for the fragmentation of the output into the correct number of candidates. This methodology was defined as Fragmentation of SIGRS Predictions (FraPS) and the adapted algorithm and script are available in Additional file 11: Figure S1. To improve the quality of the predicted sRNAs, the free energy of the RNA secondary structures was computed by RNAfold from the Vienna package [57] and normalized by the length of each sequence.

In order to support the evidence for the sRNAs predicted by SIGRS, the RNAspace pipeline [54] was used as a second method for sRNA prediction. The same input files of the set of known ncRNA genes and masked CDSs from the genomes described above were used.

### In silico analysis of gene targets

The novel sRNAs identified were analyzed by the Interacting RNA (IntaRNA) and RNAplex packages in order to find target genes for sRNA interaction. The IntaRNA software uses free energy of hybridation, target site accessibility and the presence of a seed to determine an RNA-RNA interaction [22]. RNAplex was designed to quickly find possible hybridization sites for a query RNA in large RNA databases, using a slightly different energy model that reduces the computational time. It also has a length penalty that allows to focus the target search on short highly stable interactions. The input for both IntaRNA and RNAplex was the set of sRNAs, and the targets used were all CDSs from each of the three species: *M. hyopneumoniae*, *M. flocculare* and *M. hyorhinis*. The input sequences to screen for the targets comprised 150 nucleotides upstream of the start codon until 150 nucleotides downstream start codon of each annotated gene.

To consider an interaction as positive, an energy threshold of −13 kcal/mol was set as follows. The free energies of the 390 validated interactions from the sRNATarBase 2.0 [58] were computed with IntaRNA. A regression was then calculated and plotted between the GC content and the free energy of each interaction. The GC% of *M. hyopneumoniae* is 28.5 %, of *M. flocculare* 28.9 %, and of *M. hyorhinis* 25.5 %. To effectively set a threshold, the mean of the three GC percentages was calculated and the corresponding energy value of −13 kcal/mol was taken as the threshold. The resulting predictions of both tools were compared to find ncRNA and target predictions shared by or specific to the two tools.

### Experimental analysis of the predicted M. hyopneumoniae sRNAs

#### Culture conditions and RNA isolation

*Mycoplasma hyopneumoniae* strain 7448 was subjected to three different culture conditions. In the standard condition, bacteria were grown in 40 ml Friis broth [59] at 37 °C for 24 h with gentle agitation in a roller drum. A heat shock stress condition was performed by incubation of the standard cultures (after the 37 °C for 24 h) at 30 °C for 2 h, and then shifting to 42 °C for 30 min [50]. Finally, the oxidative stress condition was obtained by addition of hydrogen peroxide (1 %) to the standard cultures followed by incubation at 37 °C for 15 min according to Schafer et al. [51].

Cells were pelleted by centrifugation at 3360 × *g* for 20 min. Total RNA was isolated with TRIzol® Reagent (Invitrogen) following the manufacturer’s instructions including DNaseI digestion with 13U of DNase I (Fermentas). Absence of DNA in the RNA preparations was monitored by PCR assays. The extracted RNA was analyzed by gel electrophoresis and quantified in the Qubit™ system (Invitrogen).

#### Stem loop reverse transcription PCR

Primers were chosen to enable the amplification of full-length predicted sRNA sequences. The designed specific stem-loop RT primer and forward primer were performed according to Chen et al. [60] searching for a target transcription at a specific predicted position of each sRNA (see example in Fig. 1a). The specificity of the stem-loop RT primers was conferred by a nine to ten nucleotide extension at the 3′ end. Primer design was performed on the *M. hyopneumoniae* 7448 genome using the Primer3 Program [61] and the sequences are described in Additional file 12: Table S12. The primer extension is a reverse complement of the last 10 nucleotides at the 3′ end of the sRNA (Fig. 1b) followed by specific forward primers.

Reverse transcriptase reactions containing 300 ng of total RNA and 10 mM deoxynucleotide triphosphates (dNTPs) were heated at 65 °C for 5 min and then incubated on ice for 2 min. ImProm II™ (Promega Inc) 5× reaction buffer (1×), 3 mM of MgCl_2_, 10 pmol of each stem–loop RT primer (Additional file 12: Table S12) and 1 μl of of ImProm II™ Reverse Transcriptase were then added to a total volume of 20 μl. The reaction mixture was incubated for 30 min at 16 °C, followed by pulsed RT of 60 cycles at 30 °C for 30 s, 42 °C for 30 s and 50 °C for 1 s, and then by reverse transcriptase inactivation at 85 °C for 5 min. Negative control was prepared in parallel, differing only by the absence of reverse transcriptase.

An end-point PCR was performed to amplify the RT product using an sRNA-specific forward primer and the universal reverse primer (Fig. 1 and Additional file 12: Table S12). GoTaq DNA Polymerase (5U – Promega Inc) was used with the following cycling parameters: 94 °C for 2 min, then 35 cycles at 94 °C for 15 s, melting temperature (T_m_) ranging from 55 to 60 °C for 1 min (T_m_s are indicated in Additional file 12: Table S12), 72 °C for 30 s, and a final step at 72 °C for 7 min. The amplification products were visualized on a 2 % agarose. The reactions were performed in experimental triplicates.

## Additional files

Additional file 1: Table S1. Small RNA (sRNA) predicted in *Mycoplasma hyopneumoniae* 7448. (XLSX 54 kb)
Additional file 2: Table S2.
*Mycoplasma hyopneumoniae* sRNAs sequence and features predicted. (XLSX 15 kb)
Additional file 3: Table S3. Transcriptional analysis of the predicted *M. hyopneumoniae* sRNAs from different culture cultivations. (XLSX 69 kb)
Additional file 4: Table S4. Small RNA (sRNA) prediction in *Mycoplasma flocculare* ATCC 27716. (XLSX 53 kb)
Additional file 5: Table S5.
*Mycoplasma flocculare* sRNAs sequence predicted. (XLSX 51 kb)
Additional file 6: Table S6. Small RNA (sRNA) prediction in *Mycoplasma hyorhinis* HUB-1. (XLSX 52 kb)
Additional file 7: Table S7.
*Mycoplasma hyorhinis* sRNAs sequence predicted. (XLSX 51 kb)
Additional file 8: Table S8. Target prediction for the sRNAs from *M. hyopneumoniae*. (XLS 17737 kb)
Additional file 9: Table S9. Target prediction for the sRNAs from *M. flocculare*. (XLS 1863 kb)
Additional file 10: Table S10. Target prediction for the sRNAs from *M. hyorhinis*. (XLS 1925 kb)
Additional file 11: Figure S1. Scripts perl. (PDF 369 kb)
Additional file 12: Table S12. Primers sequence from *M. hyopneumoniae* genome. (XLS 25 kb)
