# Supplementary material for: Mycoplasma non-coding RNA: identification of small RNAs and targets
Source: BMC Genomics. 2016 Oct 25;17(Suppl 8):743. doi: 10.1186/s12864-016-3061-z (PMC5088518; doi:10.1186/s12864-016-3061-z)
Supplement: Additional file 11: Figure S1. — Scripts perl. (PDF 369 kb) [file 12864_2016_3061_MOESM11_ESM.pdf]

# Scripts Perl

## B.1 esearch2id.pl

```
1  #!/usr/bin/perl -w
2  use strict;
3  use LWP::Simple;   #Função get
4
5  #Definido a busca
6  my $query = "mollicutes+AND+complete[status]";
7
8  #Montando a URL do esearch
9  my $base = "http://eutils.ncbi.nlm.nih.gov/entrez/eutils/";
10 my $url = $base . "esearch.fcgi?db=genome&term=$query&retmax=100000&
       usehistory=y";
11
12 #Armazenando os resultados da busca
13 my $output = get($url);
14
15 #Extraindo os IDs
16 my @a_ids;
17 while ($output =~ /<Id>(\d+)<\/Id>/sg){
18    push(@a_ids, $1);
19 }
20
21 #Extraindo as porcentagens GC de cada organismo
22 #referente a um ID e salvando-as num arquivo externo
```

```
23 my $species;
24 my $n;
25 my $gc;
26
27 open (EXF, ">ATrichMollicutes.txt");
28
29 foreach my $id (@a_ids){
30   $n = 0;
31   $gc = 0;
32
33   $url = "http://www.ncbi.nlm.nih.gov/genome/genomes/" . $id;
34   $output = get($url);
35   my @a_output = split("align=", $output);
36   if ($a_output[0] =~ /<H1 STYLE=.*>(.*)<¥/H1><table/){
37     $species = $1;
38   $species =~ s/*//g;39
     }
40   else {
41     print "It wasn't possible to find the species' name¥n";
42   $species = "Species¥' name in strange format (Id: $id.)";43
     }
44   for (my $i=11; $i<scalar @a_output; $i+=11){
45     if ($a_output[$i] =~ /"center">(¥d+¥.*¥d*)<¥/td><td/){
46       $n++;
47       $gc += $1;
48     }
49   }
50
51   print EXF ("$species¥t".$gc/$n."¥n");
52 }
53
54 exit(1);
```

## B.2 mask_anotation.pl

```
1  #!/usr/bin/perl -w
2  use strict;
3
4  #Script para um genoma no formato FASTA com as suas
5  #respectivas regiões anotadas mascaradas. Cada
6  #nucleotídeo associado a uma anotação será substituído
7  #por 'X'. As IGRs encontradas também são salvas em um
8  #arquivo externo.
9
10 unless ($ARGV[0] && $ARGV[1]){
11   print ("Three arguments requierds: (i) genome in fasta format; (ii)
         genome annotation in genbank format; (iii) header for igrs file
       ¥n");
12 }
13
14 #Arquivo para a saída do log
15 open (EXF0, ">mask-annotation.log");
16 print EXF0 ('##Inputs info¥n$ARGV[0]¥n$ARGV[1]¥n");
17
18 #Ler o arquivo do genbank e salvar as coordenadas dos
19 #nucleotídios a serem mascarados.
20 my @a_both;
21
22 open (FILE, $ARGV[1]);
23 while (my $line = <FILE>){
24   if ($line =~ /CDS¥s+(¥d+)¥.¥.(¥d+)/){
25     push(@a_both, $1);
26   push(@a_both, $2);
27   }
28   elsif ($line =~ /CDS¥s+complement¥((¥d+)¥.¥.(¥d+)¥)/){
29     push(@a_both, $1);
30   push(@a_both, $2);
31   }
32 }
```

```
33 close FILE;
34
35 if (scalar(@a_both) % 2 != 0){
36   print ("Something went wrong...¥n");
37 }
38 else{
39   print EXF0 ("¥n##Both strands info¥nNumber of genes masked = ".(
         scalar(@a_both)/2)."¥n");
40 }
41
42 #Ler o arquivo fasta e extrair o genoma
43 my $genome = "";
44 my $head;
45
46 open (FILE, $ARGV[0]);
47 while (my $line = <FILE>){
48   if ($line =~ /^>/){
49     chomp $line;
50     $head = $line;
51     next;
52   }
53
54   chomp $line;
55   $genome .= $line;
56 }
57 close FILE;
58
59 #Abrindo arquivo para salvar as IGRs
60 open (EXF1, ">sequence.igrs.fasta");
61 my $igr = 0;
62
63 #Criando fita neutra
64 my $original = 1;
65 my $segment;
66 my $i = 0;
```

```
67 my $start;
68 my $end;
69 my $nucsMasked = 0;
70 my $maskedSeq;
71 my $sobreposition = 0;
72 my $smallGene = 0;
73
74 if ($a_both[0] > 1){
75   $start = 0;
76   $end = $a_both[0]-1;
77 }
78 else{
79   $original *= -1;
80   $i++;
81
82   my $start = $a_both[0]-1;
83   my $end = $a_both[1]-1;
84 }
85
86 if ($original == 1){
87   $segment = substr($genome,$start,($end-$start));
88   $igr++;
89   print EXF1 (">IGR $igr|$ARGV[2] $start..$end¥n$segment¥n");
90 }
91 else{
92   $segment = "X" x ($end-$start+1);
93   $nucsMasked += length($segment);
94 }
95 $maskedSeq .= $segment;
96 $original *= -1;
97
98 for ($i = $i; $i<scalar(@a_both)-1; $i++){
99   my $start = $a_both[$i]-1;
100  my $end = $a_both[$i+1]-1;
101
```

```
102     if ($end <= $start){
103       $a_both[$i+1] = $a_both[$i]+1;
104       $original *= -1;
105       $sobreposition++;
106       next;
107     }
108     if ($original == 1){
109       $start++;
110       $segment = substr($genome,$start,($end-$start));
111       $igr++;
112       print EXF1 (">IGR $igr|$ARGV[2] $start..$end¥n$segment¥n");
113     }
114     else{
115       $segment = "X" x ($end-$start+1);
116       $nucsMasked += length($segment);
117       if (($end-$start+1) <= 300){
118         $smallGene++;
119       }
120     }
121     $maskedSeq .= $segment;
122     $original *= -1;
123 }
124
125 $start = $a_both[$i];
126 $end = length($genome);
127 $segment = substr($genome,$start,($end-$start));
128 if ($segment ne ""){
129     $igr++;
130     print EXF1 (">IGR $igr|$ARGV[2] $start..$end¥n$segment¥n");
131 }
132 $maskedSeq .= $segment;
133
134 my @a_maskedSeq = split("",$maskedSeq);
135 undef $maskedSeq;
136
```

```
137 open (EXF, ">sequence.masked.both.fa");
138 print EXF ($head."; both strands masked for annotated genes¥n");
139 $segment = "";
140 for (my $i=0; $i<scalar(@a_maskedSeq); $i++){
141   $segment .= $a_maskedSeq[$i];
142   if (length $segment == 70){
143     print EXF ("$segment¥n");
144     $segment = "";
145   }
146 }
147 print EXF ("$segment¥n¥n");
148 close EXF;
149
150 print EXF0 ("Initial genome size = ".length($genome)." nt¥
      nNucleotides masked = $nucsMasked nt (".(100*$nucsMasked/length(
      $genome))."% of initial)¥n");
151 print EXF0 ("Events of gene overlap = $sobreposition¥nSmall genes
      found (<=300 nt) = $smallGene¥n");
152
153 exit (1);
```

## B.3 FraPS.pl

```
1  #!/usr/bin/perl -w
2  use strict;
3
4  #Program to optimize the fragmentation of the SIGRS predictions
5  #based in the annotation covereage of known ncRNA genes
6
7  #Inputs
8  #0 SIGRS scoring file
9  #1 SIGRS ncRNA annotated predictions fasta file
10 #2 SIGRS ncRNA unannotated predictions fasta file
11
12 my @a_seq;
13 my @a_scores;
14 my @a_numericSeq;
15 my %h_seqs;
16
17 #Creation of scoring matrix (only for M1 predictions)
18
19 for my $j(0 .. 3){
20    for my $k (0 .. 3){
21    $a scores[$j][$k] = 0;22
      }
23 }
24
25 #A C G T
26 my $i =0;
27 open(FILE, $ARGV[0]); #Open SIGRS scoring file
28 while(my $line = <FILE>){
29    if ($line =~ /^#This is the M1-model scores$/)
30       $line = <FILE>;
31       $line = <FILE>;
32       do{
33          #Saving the scores in the matrix
34          chomp $line;
```

```
35          my @a_line = split(/¥s+/, $line);
36          $a_scores[$i][0] = $a_line[1];
37          $a_scores[$i][1] = $a_line[2];
38          $a_scores[$i][2] = $a_line[3];
39          $a_scores[$i][3] = $a_line[4];
40          $line = <FILE>;
41          $i++;
42      } until ($i == 4);
43    }
44 }
45 close FILE;
46
47 #Open log file to report information
48 open (LOG, ">splitPredictions.log");
49 print LOG ('##Split predictions call¥nperl split_predictions.pl @ARGV
       ¥n");
50
51 #Open SIGRS annotated predictions FASTA file
52 open(FILE,$ARGV[1]);
53 #Open file to save the annotated cumulative scoring sequences
54 open(EXF1, ">scoringMap.annotated.cumulativeResult");
55
56 my $id;
57 my $start;
58 my $end;
59 my @a_annotatedGenes;
60 my ($totalAnnotNucs, $totalLength);
61
62 while(my $seq = <FILE>){
63    chomp $seq;
64
65    #Getting annotated predictions info and the position(s) of the gene(s) where it maps
66    if($seq =~ /^>(.+)¥s+(¥d+)¥s+(¥d+)¥s+annotated¥((.+)¥)$/){
67       $id = $1;
68       $start = $2;
```

```
69      $end = $3;
70      my $annot = $4;
71      $annot =~ s/¥s,¥s/,/g;
72      @a_annotatedGenes = split (",", $annot);
73      next;
74    }
75
76    #Saving annotated gene info and preparing the sequence for cumulative score calculation
77    $h_seqs{$id}{*seq*} = $seq;
78    $h_seqs{$id}{*start*} = $start;
79    $h_seqs{$id}{*end*} = $end;
80    @{$h_seqs{$id}{*annot*}} = @a_annotatedGenes;
81    $h_seqs{$id}{*annotPerc*} = annotation_percentage($id);
82    $totalLength += length $h_seqs{$id}{*seq*};
83    @a_numericSeq = ();
84    @a_seq = split(**, $seq);
85
86    print EXF1 (">$id $start $end¥n");
87
88    #Actually calculating the cumulative scores and saving them in the output
89    #file for graph plotting
90    my $sumScores = 0;
91    my $prev = $a_seq[0];
92    for (my $i = 1; $i < scalar(@a_seq); $i++){
93      my $nuc = $a_seq[$i];
94      $prev =~ tr/ACGT/0123/;
95      $nuc =~ tr/ACGT/0123/;
96      $sumScores += $a_scores[$prev][$nuc];
97      push(@a_numericSeq, $a_scores[$prev][$nuc]);
98      print EXF1 ("$sumScores ");
99      $prev = $a_seq[$i];
100   }
101
102   print EXF1 ("¥n");
103   #Saving cumulative score
```

```
104     @{$h_seqs{$id}{*num*}} = @a_numericSeq;
105 }
106 close FILE;
107 close EXF1;
108
109 #Begining of otimization process. 'K' is the varying parameter that affects garbage percentage
110 #and unwanted exclusion of annotated nucleotides.
111 my %h_newSeqs;
112 my %h_bestSeqs;
113 my $bestEuclidianDistance = 100000;
114 my $euclidianDistance;
115 my $bestK;
116 my $garbageProportion;
117 my $bestGarbageProportion;
118 my $k;
119 for ($k = 0.01; $k < 1; $k+=0.01){
120   %h_newSeqs = ();
121
122    foreach my $id (keys %h_seqs){
123      processSequence($id, *annotated*);
124    }
125    ($euclidianDistance, $garbageProportion) = fitness(\%h_newSeqs);
126    if ($euclidianDistance >= 0 and $euclidianDistance <
         $bestEuclidianDistance){
127      $bestEuclidianDistance = $euclidianDistance;
128      $bestGarbageProportion = $garbageProportion;
129      $bestK = $k;
130      %h_bestSeqs = %h_newSeqs;
131    }
132 }
133
134 my $finalTotalLength = 0;
135 foreach (keys %h_bestSeqs){
136    $finalTotalLength += length ($h_bestSeqs{$_}{*seq*})
137 }
```

```
138
139 print LOG ("¥n##Annotated sequences information¥nInitial number of
        sequences = ".(scalar(keys(%h_seqs))).” ($totalLength nt)¥n");
140 print LOG ("Initial nucleotide garbage percent = ", 1-(
        $totalAnnotNucs / $totalLength), "¥n");
141 print LOG ("Best parameter k = $bestK¥nDistance from ideal case =
        $bestEuclidianDistance¥n");
142 print LOG ("Final number of sequences = ".(scalar(keys(%h_bestSeqs)))
        ." ($finalTotalLength nt)¥n");
143 print LOG ("Final nucleotide garbage percentage =
        $bestGarbageProportion¥n");
144
145 open(EXF2, ">ncRnaSIGRS.splited.fa");
146 foreach my $id (keys %h_bestSeqs){
147   print EXF2 (">$id $h_bestSeqs{$id}{*start*} $h_bestSeqs{$id}{*end
        *}¥n$h_bestSeqs{$id}{*seq*}¥n");
148 }
149 close EXF2;
150
151 #Open SIGRS unannotated predictions FASTA file
152 open (FILE, $ARGV[2]);
153 #Open file to save the unannotated cumulative scoring sequences
154 open(EXF1, ">scoringMap.unannotated.cumulativeResult");
155
156 #Reseting variables that store predictions info
157 @a_seq = ();
158 @a_numericSeq = ();
159 %h_seqs = ();
160 %h_newSeqs = ();
161 $totalLength = 0;
162
163 while(my $seq = <FILE>){
164   chomp $seq;
165
166   #Getting unannotated predictions info
```

```
167    if($seq =~ /^>(.+)¥s+(¥d+)¥s+(¥d+)$/){
168      $id = $1;
169      $start = $2;
170      $end = $3;
171      next;
172    }
173
174    #Saving unannotated gene info and preparing the sequence for cumulative score calculation
175    $h_seqs{$id}{*seq*} = $seq;
176    $h_seqs{$id}{*start*} = $start;
177    $h_seqs{$id}{*end*} = $end;
178    $totalLength += length $h_seqs{$id}{*seq*};
179    @a_numericSeq = ();
180    @a_seq = split(**, $seq);
181
182    print EXF1 (">$id $start $end¥n");
183
184    #Actually calculating the cumulative scores and saving them in the output file
185    #for graph plotting
186    my $sumScores = 0;
187    my $prev = $a_seq[0];
188    for (my $i = 1; $i < scalar(@a_seq); $i++){
189      my $nuc = $a_seq[$i];
190      $prev =~ tr/ACGT/0123/;
191      $nuc =~ tr/ACGT/0123/;
192      $sumScores += $a_scores[$prev][$nuc];
193      push(@a_numericSeq, $a_scores[$prev][$nuc]);
194      print EXF1 ("$sumScores ");
195      $prev = $a_seq[$i];
196    }
197
198    print EXF1 ("¥n");
199    #Saving cumulative score
200    @{$h_seqs{$id}{*num*}} = @a_numericSeq;
201  }
```

```
202 close FILE;
203 close EXF1;
204
205 $k = $bestK;
206
207 foreach my $id (keys %h_seqs){
208   processSequence($id, *unannotated*);
209 }
210
211 $finalTotalLength = 0;
212 foreach (keys %h_newSeqs){
213   $finalTotalLength += length ($h_newSeqs{$_}{*seq*})
214 }
215
216 print LOG ("¥n##Unannotated sequences information¥nInitial number of
      sequences = ".(scalar(keys(%h_seqs))).” ($totalLength nt)¥n”);
217 print LOG (”Final number of sequences = ”.(scalar(keys(%h_newSeqs))).
      ” ($finalTotalLength nt)¥n”);
218 close LOG;
219
220 open(EXF2, ”>ncRnaSIGRS_unannotated_merged.splited.fa”);
221 foreach my $id (keys %h_newSeqs){
222   print EXF2 (”>$id $h_newSeqs{$id}{*start*} $h_newSeqs{$id}{*end*}¥
        n$h_newSeqs{$id}{*seq*}¥n”);
223 }
224 close EXF2;
225
226 #
227
228 sub fitness{
229   my $refNewSeqs = shift;
230   my ($annotNucs, $length);
231   my $euclidianDistance;
232
233   foreach my $newSeq (keys %$refNewSeqs){
```

```
234     $length += length ($$refNewSeqs{$newSeq}{*seq*});
235
236     foreach my $gene (@{$$refNewSeqs{$newSeq}{*annot*}}){
237     my @a_gene = split (" ", $gene);
238     my ($start, $end);
239
240     #Computing where the overlap starts
241     if ($$refNewSeqs{$newSeq}{*start*} <= $a_gene[0]){
242       $start = $a_gene[0];
243     }
244     else{
245       $start = $$refNewSeqs{$newSeq}{*start*};
246     }
247
248     #Computing where the overlap ends
249     if ($$refNewSeqs{$newSeq}{*end*} <= $a_gene[1]){
250       $end = $$refNewSeqs{$newSeq}{*end*};
251     }
252     else{
253       $end = $a_gene[1];
254     }
255
256     $annotNucs += ($end-$start+1);
257     }
258   }
259   if (($length - $totalLength) != 0){
260     $euclidianDistance = sqrt((($annotNucs-$totalAnnotNucs)/($length-
          $totalLength))**2 + (1-($annotNucs/$length))**2);
261     print ("$k ".($annotNucs-$totalAnnotNucs)/($length-$totalLength).
          " ".(1-($annotNucs/$length))." ".$euclidianDistance."¥n");
262   }
263   else{
264     $euclidianDistance = -1;
265   }
266   return ($euclidianDistance, (1-($annotNucs/$length)));
```

```
267 }
268
269 sub inherit_overlap_gene{
270   my ($newId, $id) = @_;
271   foreach my $gene (@{$h_seqs{$id}{*annot*}}){
272     my @a_gene = split (" ", $gene);
273     my $start = $h_newSeqs{$newId}{*start*};
274     my $end = $h_newSeqs{$newId}{*end*};
275
276     if ($end < $a_gene[0]){
277       last;
278     }
279     elsif ($start == $a_gene[0]
280         and $end == $a_gene[1]){
281         push (@{$h_newSeqs{$newId}{*annot*}}, $gene);
282     }
283     elsif ($start <= $a_gene[0]
284         and $end >= $a_gene[1]){
285         push (@{$h_newSeqs{$newId}{*annot*}}, $gene);
286     }
287     elsif ($a_gene[0] <= $start
288         and $a_gene[1] >= $end){
289         push (@{$h_newSeqs{$newId}{*annot*}}, $gene);
290     }
291     elsif ($start <= $a_gene[0]
292         and $a_gene[0] <= $end){
293         push (@{$h_newSeqs{$newId}{*annot*}}, $gene);
294     }
295     elsif ($a_gene[0] <= $start
296         and $start <= $a_gene[1]){
297         push (@{$h_newSeqs{$newId}{*annot*}}, $gene);
298     }
299   }
300
301 }
```

```
302
303 #Maximum subsequence low quality detection method
304 sub processSequence {
305
306     my ($id, $annot) = @_;
307
308   my @values = @{$h_seqs{$id}{'num'}};
309     my $sequenceSize = @values;
310     my $subseqNum = 1;
311     my $newId;
312
313     #Maximum global information
314     my $maxGlobal = 0;
315     my $maxGlobalBegin = 0;
316     my $maxGlobalEnd = 0;
317
318     #Maximum suffix information
319     my $maxSuffix = 0;
320     my $maxSuffixBegin = 0;
321
322     #Auxiliar for the cummulated sum
323     my $auxSum = 0;
324
325     #Tolerance for decreasing values in subsequences
326     my $tolerance = 0;
327   foreach my $value (@values) {
328     $tolerance += $value;
329   }
330   $tolerance *= $k;
331
332   #Minimum ncRNA length (reference Christine)
333   my $minimumLength = 70;
334
335     #Process the whole sequence
336     for (my $i = 0; $i < $sequenceSize; $i++) {
```

```
337
338         #Add the next value to the cumsum
339         $auxSum += $values[$i];
340
341         if ($auxSum >= $maxGlobal) {
342           #Update maxGlobal and maxSuffix
343           $maxSuffix = $auxSum;
344           $maxGlobal = $maxSuffix;
345           $maxGlobalBegin = $maxSuffixBegin;
346           $maxGlobalEnd = $i + 1;
347         } else {      #Started to decrease
348
349           if ($auxSum <= 0) {
350             #If the cumsum is smaller than zero, reset the maximum suffix information
351             $maxSuffix = 0;
352             $maxSuffixBegin = $i + 1;
353             #Also, reset the auxiliar variable
354             $auxSum = 0;
355           } else {
356
357             #Update the maximum suffix
358             $maxSuffix = $auxSum;
359
360             #Simple criterium : if the difference between maximum global
361             #and maximum suffix is bigger than the tolerance established,
362             #output the current maximum global and start to search for a
363             #new region.
364             if ($maxGlobal - $maxSuffix > $tolerance
365               and $maxGlobalEnd - $maxGlobalBegin + 1 > $minimumLength)
                    {
366               $newId = $id.*.*.$subseqNum;
367               $h_newSeqs{$newId}{*seq*} = substr($h_seqs{$id}{*seq*},
                      $maxGlobalBegin,($maxGlobalEnd - $maxGlobalBegin + 1))
                      ;
```

```
368             $h_newSeqs{$newId}{*start*} = $h_seqs{$id}{*start*} +
                    $maxGlobalBegin ;
369             $h_newSeqs{$newId}{*end*} = $h_newSeqs{$newId}{*start*}
                     +($maxGlobalEnd - $maxGlobalBegin);
370             if ($annot eq *annotated*){
371               inherit_overlap_gene($newId, $id);
372             }
373             $subseqNum++;
374             $maxGlobal = 0;
375             $maxGlobalBegin = $i;
376             $maxSuffix = 0;
377             $maxSuffixBegin = $i;
378             $auxSum = 0;
379           }
380
381         }
382
383       }
384
385     }
386
387     #Print the last region
388     if ($subseqNum == 1){
389       $h_newSeqs{$id} = $h_seqs{$id};
390     }
391     elsif ($maxGlobalEnd - $maxGlobalBegin > $minimumLength){
392     $newId = $id.*.*.$subseqNum;
393       $h_newSeqs{$newId}{*seq*} = substr($h_seqs{$id}{*seq*},
            $maxGlobalBegin,($maxGlobalEnd - $maxGlobalBegin + 1));
394     $h_newSeqs{$newId}{*start*} = $h_seqs{$id}{*start*} +
          $maxGlobalBegin ;
395     $h_newSeqs{$newId}{*end*} = $h_newSeqs{$newId}{*start*} + (
          $maxGlobalEnd - $maxGlobalBegin);
396     if ($annot eq *annotated*){
397       inherit_overlap_gene($newId, $id);
```

```
398     }
399     }
400
401 }
402
403
404 sub annotation_percentage{
405   my $length = length ($h_seqs{$id}{*seq*});
406   my $overlap = 0;
407
408   foreach my $gene (@{$h_seqs{$id}{*annot*}}){
409     my @a_gene = split (" ", $gene);
410     my ($start, $end);
411
412     #Computing where the overlap starts
413     if ($h_seqs{$id}{*start*} <= $a_gene[0]){
414       $start = $a_gene[0];
415     }
416     else{
417       $start = $h_seqs{$id}{*start*};
418     }
419
420     #Computing where the overlap ends
421     if ($h_seqs{$id}{*end*} <= $a_gene[1]){
422       $end = $h_seqs{$id}{*end*};
423     }
424     else{
425       $end = $a_gene[1];
426     }
427
428     $overlap += ($end-$start+1);
429   }
430
431   $totalAnnotNucs += $overlap;
432   return ($overlap / $length);
```

```
433 }
```
